# Effects of intermittent fasting on cognitive health and Alzheimer’s disease

**DOI:** 10.1093/nutrit/nuad021

**Published:** 2023-04-12

**Authors:** Alby Elias, Noushad Padinjakara, Nicola T Lautenschlager

**Affiliations:** Academic Unit for Psychiatry of Old Age, Department of Psychiatry, The University of Melbourne, North-Western Mental Health, Melbourne Health, Victoria, Australia; Department of Endocrinology and Metabolic Medicine, South Warwickshire University NHS Foundation Trust, Coventry, United Kingdom; Academic Unit for Psychiatry of Old Age, Department of Psychiatry, The University of Melbourne, North-Western Mental Health, Melbourne Health, Victoria, Australia

**Keywords:** Alzheimer’s disease, dementia, intermittent fasting, risk

## Abstract

**Objective:**

Caloric restriction by intermittent fasting produces several metabolic changes, such as increased insulin sensitivity and use of ketone bodies as energy sources. In humans, intermittent fasting has been studied in hypertension, diabetes, and related conditions, but, to date, not as a strategy to reduce the risk of emergent dementia. In this scoping review, the relevance of intermittent fasting as a potential preventive intervention for Alzheimer’s dementia is explored.

**Background:**

The beneficial effects of calorie restriction have been documented in animals and humans. Decreased oxidative stress damage and attenuated inflammatory responses are associated with intermittent fasting. These changes have a favorable impact on the vascular endothelium and stress-induced cellular adaptation.

**Results:**

Physiological alterations associated with fasting have profound implications for pathological mechanisms associated with dementias, particularly Alzheimer’s disease. Compared with ad libitum feeding, caloric restriction in animals was associated with a reduction in β-amyloid accumulation, which is the cardinal pathological marker of Alzheimer’s disease. Animal studies have demonstrated synaptic adaptations in the hippocampus and enhanced cognitive function after fasting, consistent with these theoretical frameworks. Furthermore, vascular dysfunction plays a crucial role in Alzheimer’s disease pathology, and intermittent fasting promotes vascular health.

**Conclusions:**

These observations lead to a hypothesis that intermittent fasting over the years will potentially reverse or delay the pathological process in Alzheimer’s disease.

## INTRODUCTION

A dramatic increase in the human lifespan in the past century has not translated to an increase in healthy years of life (health span), especially in older age.^1^ It is estimated that the proportion of people older than 65 years will double from 8.5% to 17% by 2050.^2^ Such a demographic transition poses extraordinary challenges for the medical community and policy makers, given that older age is one of the strongest risk factors for cardiovascular diseases, cancers, metabolic syndrome, and neurodegenerative syndromes, including dementia.^3–5^ The 2017 Global Burden of Diseases study identified 92 age-related diseases, and these accounted for 51.3% of all disease.^5^

The process of aging is complex and not well understood. For instance, chronological age indicates calendar-years and does not always predict biological health, changes at the cellular level, morbidity, or death.^6^ Oxidative damage is one of the most discussed mechanisms of aging.^7–9^ Accordingly, reactive oxygen and nitrogen free species that are constantly produced as the byproducts of cellular metabolism bombard the cell nucleus. The body’s antioxidant defense system normally neutralizes these species. An imbalance favoring reactive species causes cumulative cellular damage and subsequent aging over time. Also, DNA telomeric shortening is associated with aging. Caloric restriction without malnutrition has been proven to reduce the rate of cellular metabolism, the production of reactive species responsible for oxidative damage, and telomeric shortening, and hence has been proposed to have the potential to combat age-related diseases.^10–12^

### Intermittent fasting

Beneficial effects of intermittent fasting on aging have increasingly attracted interest in recent years.^13^^,^^14^ People have practiced intermittent fasting for millennia.^15^ In the preliterate society, intermittent fasting was often the norm when food was scarce. The past few decades have witnessed a renewed interest in intermittent fasting and a concerted research effort focusing on the various health benefits of fasting. Studies have addressed both clinical and biochemical changes associated with fasting. These studies were conducted in animals to a large extent, but human trials have also been conducted recently.^13^^,^^15^

There is no uniform definition of intermittent fasting. Fasting is characterized by longer intervals between meals without enduring nutritional deficiency.^16^ It is a dietary pattern consisting of cycles of fasting for a duration varying from 12 hours to 24 hours alternating with periods of normal food intake. Thus, it is not related to a particular type of diet; it is a pattern of eating. Different regimens of intermittent fasting are practiced. The common patterns include 12–16 hours of daily fasting, 20–24 hours of fasting 2 days a week, or more intense patterns, such as alternate-day fasting.^13^^,^^17^^,^^18^ Prolonged food deprivation beyond 24 hours is starvation and considered different from fasting, given its unique and harmful biochemical changes. Ad libitum feeding is the consumption of food without restrictions.

### Biology of intermittent fasting

Glucose is the primary energy source for humans. Glucose metabolism is time dependent in that it is a function of time since the last meal. In humans, the blood glucose level falls shortly after consumption of a carbohydrate meal. Depending on the amount of glycogen stored in the liver and the energy expenditure after that, the glycogen level will be diminished and fat metabolism becomes the energy source, through the production of ketone bodies during the 12–36 hours after carbohydrate intake.^18^^,^^19^ Several organs, including the brain, can use ketone bodies for energy requirements.^13^^,^^16^ This kind of metabolic switch, from glucose to ketone bodies, is the most characteristic metabolic feature of fasting.^18^ Ketone bodies that are naturally produced during fasting are only minimally increased in the first 24 hours after the last meal.^19^^,^^20^

The key mechanism of benefits of fasting is through heightened insulin sensitivity.^12^^,^^21^^,^^22^ It is well known that insulin sensitivity is decreased in type 2 diabetes mellitus.^23–26^ Insulin resistance promotes atherosclerosis and subsequent vascular diseases.^27^^,^^28^ In addition to the effect on insulin level and sensitivity, fasting has been associated with reduced lipids and blood pressure levels in controlled clinical trials in humans.^29–34^ These changes exert highly beneficial effects on blood vessels. In a nutshell, intermittent fasting promotes vascular health.

At a molecular level, fasting is associated with optimization of cellular metabolism.^15^^,^^16^^,^^35^ This is evident in alterations in thyroid hormones and the amount of free oxygen radicals.^10–12^ Oxygen free radicals are produced as the byproducts of normal oxidative metabolism. Free radicals have the potential to cause genome mutations and diseases. During fasting, metabolism and protein synthesis are temporarily reduced and, hence, the formation of free radicals is attenuated.^36^ Also, cells undergo stress response and adaptation during fasting. These changes include enhanced antioxidant mechanisms, DNA repair, removal of cellular waste products, or autophagy.^13^^,^^37^ Upon restoration of feeding and the glucose supply, cells regain homeostasis and thus become more stress resistant. Brain cells in animals maintained on intermittent fasting exhibited improved function and adaptive response to metabolic, traumatic, and oxidative stress.^36^

### Pathology of Alzheimer’s disease

Before discussing fasting in Alzheimer’s disease (AD), it is worthwhile to recapitulate the disease pathology (Table 1). Extracellular β-amyloid plaques and intracellular tangles are the characteristic neurological lesions in AD.^38^^,^^39^ The neurofibrillary tangles are composed of abnormally phosphorylated tau proteins, which are regarded as the downstream effect of excess β-amyloid.^40^^,^^41^ Although early-onset AD arises from the excess production of β-amyloid, late onset AD, the most common type, results from decreased clearance of β-amyloid from the brain.^42^^,^^43^ Apolipoprotein E ε4 is associated with impaired clearance of β-amyloid and hence contributes to an increased risk of dementia.^44–46^

**Table 1:** Putative mechanisms of benefits of intermittent fasting in Alzheimer’s disease

Pathological factors in Alzheimer’s disease	Findings
β-Amyloid and tau deposition	Increase in ketone bodies as a result of fasting may reduce β-amyloid level. Caloric restriction reduced both β-amyloid level and tau. Fasting may not reduce the amyloid level in cases of Alzheimer's disease with a strong genetic load.
Cerebrovascular diseases	Enhanced vascular integrity after fasting Diminished impact of stroke Potentially synergetic effects of cardiovascular and cerebrovascular health
Inflammation	Inflammation may play a key role in Alzheimer’s disease. Intermittent fasting is associated with reduced levels of inflammation.

An epidemiological and mechanistic association exists between cerebrovascular risk factors and Alzheimer’s dementia.^47–49^ Originally proposed in 2001, the neurovascular unit in the brain received abundant interest in the following years, and recently emerged evidence suggests an important role of this vascular unit in the genesis of AD pathology.^49^^,^^50^ A neurovascular unit comprises brain endothelial cells lining the cerebral vasculature, pericytes covering capillaries, vascular smooth cells, glia, and surrounding neurons. The blood-brain barrier (BBB) lies within the neurovascular unit and regulates the transport of molecules between the brain interstitial fluid and cerebrovascular compartment. The BBB plays a critical role in the clearance of β-amyloid from the brain.^51–53^ Cerebrovascular diseases, particularly chronic small-vessel disease, lead to BBB disruption and dysfunction and act as a risk factor for AD.^54–56^ In support of this hypothesis, neuroimaging studies have documented 20% lower cerebral blood flow and reduced pulse pressure in patients with Alzheimer’s dementia compared with individuals without dementia.^57^ Postmortem data indicate BBB damage in AD.^58^ Adding to accruing evidence, authors of a recent study identified gene expressions that implicate dysregulated blood flow in AD.^59^ These findings suggest profound alterations of cerebral vasculature in AD.

Although anti-inflammatory drug trials in the setting of AD have generated inconsistent findings, and there is no definite evidence supporting their use in preventing Alzheimer’s dementia, other findings suggest a role of inflammation in the pathogenesis of AD.^60–62^ β-Amyloid and tau tangles are necessary but not sufficient in themselves in the causation of Alzheimer’s dementia. Robust evidence supports the hypothesis of sustained inflammation as a driving factor for progression of AD to clinical dementia.^63^^,^^64^ This is evidenced in neuroimaging and human postmortem studies.^62^^,^^65–67^ Intriguing findings from a recent positron emission tomographic study suggest a biphasic inflammatory reaction in AD: the initial phase is caused by β-amyloid plaques and the second phase is triggered by tau tangles.^68^ β-Amyloid–induced neuroinflammation occurring in the early stage of prodromal Alzheimer’s dementia may be protective, but later in the prodromal stage, activated microglia fail to clear β-amyloid deposition, and the inflammatory response diminishes. As β-amyloid continues to increase, it triggers tau propagation through a yet unknown mechanism with which the second wave of inflammation ensues.^68^ The implication is that reducing inflammation in the appropriate stage may attenuate the disease process and either delay or prevent the onset of clinical manifestations of AD, including mild cognitive impairment.

In view of the aforementioned biological mechanisms associated with intermittent fasting and their implications for pathological processes in AD, the aim for this scoping review was to explore the potential benefits, safety, and feasibility of intermittent fasting in reducing the risk of AD.

## METHOD

The search of literature included the following keywords: “intermittent fasting,” “Alzheimer’s disease,” and “dementia.” The search sources included the Medline (through PubMed), Scopus, and Embase databases; no language or time restrictions were imposed. Titles and abstracts were screened, and relevant full-article texts were extracted, reviewed, and downloaded to Endnote (Clarivate). Uncertainty was resolved through consultation among all authors.

## RESULTS

### Intermittent fasting and Alzheimer’s disease pathology

#### β-Amyloid and tau

Fasting leads to an increase in ketone bodies.^69^ The earliest evidence for the protective effect of ketone bodies against the toxic effect of β-amyloid came in 2000.^70^ Later, Versele et al^71^ demonstrated that ketone bodies augmented β-amyloid efflux across the BBB to blood in a human in vitro model. A subsequent experiment proved that compared with ad libitum feeding, caloric restriction was associated with a lower level of phospho tau in the hippocampus and β-amyloid in mice expressing dominantly inherited genes for amyloid precursor protein and presenilin I, and intermittent fasting was associated with better cognitive performance in comparison with ad libitum feeding.^72^ In animal studies, intermittent fasting was associated with reduced levels of brain β-amyloid through various mechanisms.^73^ Researchers later demonstrated that a diet containing physiological precursors of ketone bodies was associated with improved performance on learning and memory tasks in the mouse model of AD, along with a reduction in β-amyloid and hyperphosphorylated tau levels.^74^ As shown earlier, increase in ketone body levels is the metabolic signature of intermittent fasting. In line with these observations, increased risk for death resulting from focal ischemic stroke diminished in middle-aged mice undergoing intermittent fasting compared with a control group. Markers of inflammation such as cytokine levels also decreased with intermittent fasting.^75^

Studies assessing the impact of intermittent fasting on β-amyloid level have yielded conflicting results, however.^72^^,^^73^^,^^76^^,^^77^ The outcomes of alternate-day fasting varied based on study protocols and disease models. For instance, Zhang et al^73^ studied double-transgenic mice at the age of 5 months and found benefits of intermittent fasting in reducing cognitive impairment and β-amyloid level. Halagappa et al^72^ found improved cognitive performance with intermittent fasting at 17 months in a triple-transgenic mouse model but no reduction in β-amyloid compared with the control diet. Lazic et al^76^ studied a mouse model with 5 mutations and found that every-other-day feeding was associated with increased inflammatory and neurodegenerative changes without alterations in the β-amyloid level.^76^ In rats subjected to ovariectomy and β-amyloid infusion, 3-hour feeding per day intermittent fasting prevented memory loss but exacerbated bone density loss.^77^ Extrapolating these findings to humans, it could be possible that intermittent fasting may not reduce the amyloid load in cases of AD with a strong genetic load, such as familial AD. In contrast, the β-amyloid deposition in late-onset sporadic AD may be amenable to fasting.

#### Intermittent fasting and cerebrovascular diseases

Endothelial cells play a major role in the movements of ions, molecules, and cells into and out of the brain and, thus, the thromboresistance property of vessels and vascular homeostasis.^78^ Endothelial dysfunction is a broad term encompassing oxidative stress, low-grade inflammation, increased vascular tone, loss of BBB integrity, atherosclerosis, and thrombosis.^78^ Therefore, endothelial dysfunction acts as a risk factor for stroke.^79^ Human trials have shown improved endothelial function as assessed by markers of endothelial integrity, such as asymmetric dimethylarginine and cutaneous microcirculation with laser Doppler scan, in individuals after fasting.^80–82^ Prolonged fasting in mice was associated with an increase in endothelial progenitor cells and improved stroke outcome.^83^ In animal models of ischemia, intermittent fasting reduced infarct size, brain edema, the amount of neuronal loss through autophagy, and minimized apoptosis.^84^^,^^85^ Given the similar size of the vasculature in the heart and brain with the same endothelial structure and function and shared risk factors, there is a pathophysiological relationship between cardiovascular and cerebrovascular diseases.^86^ Animals maintained on intermittent fasting had improved heart rate variability, a sign of adaptive cardiovascular health.^87^ Low heart rate variability is an independent risk factor for coronary artery disease, and its presence predicts the presence of myocardial ischemia 2-fold.^88^

#### Intermittent fasting and inflammation

Intermittent fasting reduces inflammation. In a randomized controlled trial (RCT) of patients with rheumatoid arthritis, fasting was associated with improvement in symptoms of inflammation such as swelling and pain and inflammatory markers, namely, erythrocyte sedimentation rate, C-reactive protein level, and white blood cell count.^89^ These findings were replicated in subsequent trials, and authors of a meta-analysis concluded that long-term benefits followed fasting in rheumatoid arthritis.^90^ In the same vein, in a trial of participants with asthma, those who maintained intermittent fasting had reduced inflammatory markers compared with those who followed an ad libitum diet.^17^ Obesity is associated with many serious diseases, including cerebrovascular disease and AD.^91–93^ Obesity is considered a state of chronic inflammation.^94^ There is high-quality evidence for the efficacy of intermittent fasting in reducing obesity and improving cardiometabolic parameters.^95^ Inflammatory markers, such as circulating peripheral monocytes, are reduced during intermittent fasting.^96^^,^^97^ Ramadan fasting in healthy adults was followed by significant reduction in proinflammatory cytokines, tumor necrosis factor α, and the number of immune cells.^96^ In a recent RCT, levels of galaectin-3, an inflammation modulating substance, increased after 26 weeks of 24-hour water-only fasting twice weekly for 4 weeks followed by once weekly for 22 weeks.^98^

### Benefits of intermittent fasting in cognitive function and Alzheimer’s disease: empirical evidence

#### Animal studies

Fasting is an evolutionarily conserved adaptive behavior in the animal kingdom.^13^^,^^16^ Animals, including humans, maintain a high level of cognitive function during fasting, which is adaptive upon facing food scarcity.^16^ In line with these general presumptions, empirical evidence suggested that animals undergoing long-term intermittent fasting had improved motor coordination, learning, and consolidation of memory in comparison with ad libitum–fed animals.^99^^,^^100^ Furthermore, dietary restriction in animals was followed by an increase in newly generated cells in the dentate gyrus of the hippocampus and the expression of brain-derived neurotrophic factor (BDNF) and neurotrophin-3.^101^ Intriguingly important is the role of BDNF given that the genes coding for BDNF and its receptor are present in vertebrates, not in worms, flies, or species lower on the evolutionary ladder.^16^ In this way, intermittent fasting is arguably an intervention targeting the disease process from multiple angles in Alzheimer’s dementia.

In mouse models, intermittent fasting from the age of 3 months was associated with improved exploratory behavior and performance in both the goal latency and probe trials when assessed at the age of 17 months in comparison with mice allowed ad libitum feeding.^72^ In a similar study, mice maintained on intermittent fasting had enhanced learning and consolidation processes that were associated with facilitation of synaptic plasticity in the hippocampus, in comparison with mice allowed ad libitum feeding.^100^ In mice subjected to alternate-day intermittent fasting, learning and memory performance were better than in control mice fed a high-fat diet.^102^ Enhanced cognitive performance was associated with a thickened CA1 pyramidal cell layer in the hippocampus. Animal research suggests that BDNF contributed to neurogenesis induced by dietary restriction.^48^ Contrary to other findings, authors of one study observed worsening of cerebral inflammation and cognitive function without significant changes in β-amyloid after 4 months of alternate-day fasting in mice.^76^ In this study, alternate-day fasting began at the age of 2 months.

#### Human trials

The effects of short-term fasting lasting a month have been typically studied in humans after Ramadan fasting. These real-world, prospective studies have involved participants of different ages and focused on the tolerability and adverse effects of fasting, rather than long-term cognitive benefits.^103–106^ The results are heterogeneous: some findings suggest improvement in vigilance and processing speed with diurnal variation.^103–105^ Although a few studies indicated an increased daytime drowsiness during Ramadan fasting, most studies did not show such an effect.^103^ Taken together, data from these short-term studies do not suggest an overall significant adverse impact of fasting on sleep quality, daytime sleepiness, or cognitive function.^103^^,^^105^ Studies of short-term fasting not associated with religion also showed inconsistent results regarding cognitive performance, with some studies showing deficits particularly in processing speed and executive functions, whereas others showed no significant changes.^107^^,^^108^

Studies of long-term fasting have been limited in humans. Witte et al^109^ demonstrated an improvement in verbal memory scores after 3 months of caloric restriction in healthy study participants with a mean age of 60 years. Leclerc et al^110^ replicated the cognitive benefits after a longer period of regular caloric restriction for up to 24 months in a larger sample of 220 healthy participants (age range, 21–50 years). Improvement in working memory with caloric restriction was documented in comparison with an ad libitum diet.^110^ Effects specific to fasting remained unknown, however. A longitudinal study of the neuroprotective model for healthy longevity, involving older adults, followed up 99 participants with mild cognitive impairment who were older than 60 years. Of the study participants, 37 practiced intermittent fasting 2 days/week from dawn to sunset, 35 observed fasting for 12 months, and 27 did not practice fasting.^111^ The proportion of participants with successful aging (defined as being free of common chronic diseases, Mini–Mental State Examination score > 22, good functional ability, and good quality of life) was markedly higher in the regular fasting group (24.3%) compared with the nonfasting group (3.1%). This study was limited by small sample sizes.^111^ Another trial showed the beneficial impact of externally administered ketone body, medium-chain triglycerides on cognitive performance in comparison with placebo in people with mild cognitive impairment.^112^ Neurons involved in neurodegenerative diseases have common features such as mitochondrial dysfunction and oxidative damage, which result in energy crisis.^113^ These selective neurons have a high energy demand, and when glucose metabolism is compromised, ketone bodies provide alternate energy sources. It is noteworthy that ketone metabolism remains unaltered in the early stages of neurodegenerative diseases.^113^

#### Adverse effects of intermittent fasting: limitations, challenges, and barriers

Authors of a systematic review and meta-analysis observed that intermittent fasting is largely safe.^114^ Nonetheless, lack of energy, headaches, feeling cold, constipation, bad breath, lack of concentration, and bad temper have been reported.^114^ The practice of intermittent fasting needs careful preparation after considering physical and psychological health (Table 2). Because diabetes mellitus involves substantial alterations in insulin levels and sensitivity and, hence, glucose levels, safety and tolerability of intermittent fasting in this condition, particularly in those receiving insulin and oral hypoglycemic agents, warrant more trials.^115^ Although largely protective of neuronal health and neuroplasticity, fasting promoted neurodegeneration in the inherited models of amyotrophic lateral sclerosis and did not offer protection against the progression of the disease, suggesting that motor neurons involved in this disease may not be able to adapt to the stress of intermittent fasting.^116^^,^^117^ A few reports indicate that intermittent fasting could be associated with loss of lean body mass in addition to fat mass.^118^^,^^119^ Available evidence shows that loss of appendicular muscle mass, however, can be prevented, including in older people, by resistance training and incorporating protein-rich food into the meal regimens.^120–122^ Fasting may not be advisable for patients with advanced or rapidly progressing dementia. Although a high body mass index is associated with an increased risk of AD in midlife, reduced appetite, weight loss, and malnutrition may occur in the late phase of dementia.^92^^,^^123^ Likewise, recent unintentional weight loss should alert the clinician to the risk of malnutrition or emergence of new medical issues, and fasting is not recommended in such instances.

**Table 2:** Precaution and adverse effects of intermittent fasting

Condition	Complications
Type 1 and 2 diabetes mellitus	Paucity of evidence on safety Extra caution such as frequent self-monitoring of capillary glucose required. Continuous glucose monitoring and flash glucose monitoring with alarm systems are now widely available, enabling safe adoption of intermittent fasting.
Anorexia nervosa (study subjects with BMI <25)	Potential worsening of anorexia nervosa and excess weight loss
Amyotrophic lateral sclerosis	Motor neurons may be vulnerable to prolonged fasting. Enhanced neurodegeneration in the inherited form of amyotrophic lateral sclerosis.
Loss of lean body mass	Resistance training required

In 2021, authors of a Cochrane review found that intermittent fasting was superior to ad libitum diet in reducing weight, albeit not statistically significantly so.^124^ There is paucity of data on outcomes such as all-cause mortality, cardiovascular mortality, stroke, myocardial infarction, or heart failure. There was no significant difference between intermittent fasting and caloric restriction without intermittent fasting in cardiometabolic risk factors, an observation replicated in a recent trial.^125^ The evidence base indicating beneficial effects of fasting on Alzheimer’s pathology, such as β-amyloid deposition, is preliminary and derived from animal studies. Additionally, findings regarding the effect of intermittent fasting on AD pathology, such as β-amyloid deposition, are inconsistent.^72^^,^^76^^,^^126^ Human trials in which outcomes related to AD were assessed are limited.

One of the limitations in the RCTs of intermittent fasting was poor adherence to interventions.^127^^,^^128^ Self-reported adherence to an intervention regimen varied widely from 29% to 92%, with better adherence to 16 hours of daily fasting and a reminder through an app than to 5 days of normal eating and a twice-weekly calorie restriction regimen.^118^^,^^127^ Previously published reports from RCTs of intermittent fasting showed a dropout rate that varied from 18% to 34% over 12 months, whereas the dropout rate for the control groups was 26%.^11^^,^^118^^,^^127^^,^^128^ In the study by Trepanowski et al,^128^ the dropout rate was attributed more to the alternate-day fasting group than the daily fasting group. Moreover, alternate-day fasting was not superior to the daily fasting regimen.^128^ With a completion rate of 88%, Ravussin et al^11^ demonstrated the feasibility of a 2-year RCT of caloric restriction compared with an ad libitum diet in 218 people aged 21–51 years. In a meta-analysis, authors found an overall adherence of 60.5% in intervention trials for weight loss.^129^ Adherence to the interventions improved with social support and dietary intervention relative to exercise alone.

#### Intermittent fasting in the older population

Several studies investigated the effects of intermittent fasting in the older population.^109^^,^^121^^,^^130–132^ Ooi et al^111^ prospectively studied intermittent fasting in older individuals with mild cognitive impairment for 3 years and assessed the cognitive outcomes. A pilot study of 10 participants aged ≥65 years suggested that the older population could tolerate 14–18 hours of daily fasting for 4 weeks, with 84% adherence.^130^ Another randomized trial investigated the effects of a hypocaloric diet in older obese individuals for 13 weeks.^121^ In a controlled trial, 45 women older than 60 years participated in 16 hours of daily fasting for 6 weeks and achieved significant weight reduction.^131^ Response to caloric restriction in 50 healthy, normal weight to overweight older individuals was a decrease in plasma insulin and C-reactive protein levels and improved verbal memory performance at the end of 3 months.^109^ In an RCT pilot study, older male veterans successfully completed 5 days of normal eating and a twice-weekly fasting regimen that lasted for 6 months.^132^

## DISCUSSION

There are no definite treatments or preventive drugs for dementias arising from Alzheimer’s and other neurodegenerative diseases. Notably, the pathological processes in AD are complex and extend beyond β-amyloid and tau.^133^ Our current understanding of AD is likely to reflect a proximate etiology rather than the original cause. Vascular diseases play a critical role in late-onset AD. Oxygen free radicals and genomic mutations are also implicated in AD.^134^^,^^135^ In this context, it is worth considering intermittent fasting, given its favorable impact on vascular endothelium, cellular metabolism, production of oxygen free radicals, and consequent diminished risk for genome mutations.

Intermittent fasting for 12–24 hours appears to be a promising approach to reduce the risk of AD pathology and its clinical manifestation of dementia. Two sets of empirical findings support this hypothesis, one from animal studies demonstrating a favorable impact of intermittent fasting on AD pathology and the other derived from human studies showing the benefits of fasting in reducing the risk of cardiovascular disease, inflammatory conditions, and obesity, which are associated with AD pathology.^32^^,^^90^^,^^93–95^^,^^136^ This heterogeneity of pathological processes implies the need for interventions that have broad actions. Intermittent fasting may meet these requirements, with its profound and widespread cellular and metabolic effects.

Human trials of the effects of intermittent fasting on cognitive function and dementia are limited, presumably because of trial adherence and difficulties in maintaining a fasting regimen. However, other lifestyle interventions, such as exercise, were studied and found to reduce cognitive decline in older people. A YouGov poll among more than 1200 US adults in 2020 showed that 24% of the surveyed population practiced intermittent fasting at some stage in their lives, a figure consistent with systematic data from the US Healthy Minds Study.^137^^,^^138^ Reports from Australian media suggest a growing enthusiasm for the practice of intermittent fasting.^139^ Possibly enrichment trials targeting the population at risk of dementia can address the challenges in intermittent fasting trials.

## CONCLUSIONS

Intermittent fasting may be tested in clinical trials of AD for safety, feasibility, and efficacy given the broad cellular and metabolic impact of intermittent fasting that can favorably affect AD pathology from multiple angles. In particular, the beneficial effects of intermittent fasting in promoting vascular health and reducing oxidative damage provide empirical support for such trials.

## References

[1] Seals DR , Justice JN, LaRocca TJ. Physiological geroscience: targeting function to increase healthspan and achieve optimal longevity. J Physiol. 2016;594:2001–2024. 25639909 10.1113/jphysiol.2014.282665 PMC4933122

[2] He W , Goodkind D, Kowal P. *US Census Bureau, International Population Reports, P95/16-1, an Aging World: 2015*. Washington, DC: US Government Publishing Office; 2016.

[3] Niccoli T , Partridge L. Ageing as a risk factor for disease. Curr Biol. 2012;22:R741–R752. 22975005 10.1016/j.cub.2012.07.024

[4] Bishop NA , Lu T, Yankner BA. Neural mechanisms of ageing and cognitive decline. Nature. 2010;464:529–535. 20336135 10.1038/nature08983 PMC2927852

[5] Olshansky SJ , Ault AB. The fourth stage of the epidemiologic transition: the age of delayed degenerative diseases. Milbank Q. 1986;64:355–391. 3762504

[6] Henderson YO , Bithi N, Link C, et al Late-life intermittent fasting decreases aging-related frailty and increases renal hydrogen sulfide production in a sexually dimorphic manner. GeroScience. 2021;43:1527–1554. 33675469 10.1007/s11357-021-00330-4 PMC8492807

[7] Liguori I , Russo G, Curcio F, et al Oxidative stress, aging, and diseases. Clin Interv Aging. 2018;13:757–772. 29731617 10.2147/CIA.S158513 PMC5927356

[8] Selman C , Blount JD, Nussey DH, et al Oxidative damage, ageing, and life-history evolution: where now? Trends Ecol Evol. 2012;27:570–577. 22789512 10.1016/j.tree.2012.06.006

[9] Lin MT , Beal FM. The oxidative damage theory of aging. Clin Neurosci Res. 2003;2:305–315.

[10] Redman LM , Smith SR, Burton JH, et al Metabolic slowing and reduced oxidative damage with sustained caloric restriction support the rate of living and oxidative damage theories of aging. Cell Metab 2018;27:805–815.e804. 29576535 10.1016/j.cmet.2018.02.019 PMC5886711

[11] Ravussin E , Redman LM, Rochon J, et al; for the CALERIE Study Group. A 2-year randomized controlled trial of human caloric restriction: feasibility and effects on predictors of health span and longevity. J Gerontol A Biol Sci Med Sci 2015;70:1097–1104. 26187233 10.1093/gerona/glv057 PMC4841173

[12] Heilbronn LK , de Jonge L, Frisard MI, et al Effect of 6-month calorie restriction on biomarkers of longevity, metabolic adaptation, and oxidative stress in overweight individuals: a randomized controlled trial. JAMA. 2006;295:1539–1548. 16595757 10.1001/jama.295.13.1539 PMC2692623

[13] de Cabo R , Mattson MP. Effects of intermittent fasting on health, aging, and disease. N Engl J Med. 2019;381:2541–2551. 31881139 10.1056/NEJMra1905136

[14] Di Francesco A , Di Germanio C, Bernier M, et al A time to fast. Science. 2018;362:770–775. 30442801 10.1126/science.aau2095 PMC8504313

[15] Patterson RE , Laughlin GA, LaCroix AZ, et al Intermittent fasting and human metabolic health. J Acad Nutr Diet. 2015;115:1203–1212. 25857868 10.1016/j.jand.2015.02.018 PMC4516560

[16] Longo VD , Mattson MP. Fasting: molecular mechanisms and clinical applications. Cell Metab. 2014;19:181–192. 24440038 10.1016/j.cmet.2013.12.008 PMC3946160

[17] Johnson JB , Summer W, Cutler RG, et al Alternate day calorie restriction improves clinical findings and reduces markers of oxidative stress and inflammation in overweight adults with moderate asthma. Free Radic Biol Med. 2007;42:665–674. 17291990 10.1016/j.freeradbiomed.2006.12.005 PMC1859864

[18] Anton SD , Moehl K, Donahoo WT, et al Flipping the metabolic switch: understanding and applying the health benefits of fasting. Obesity (Silver Spring). 2018;26:254–268. 29086496 10.1002/oby.22065 PMC5783752

[19] Cahill GF. Jr . Starvation in man. N Engl J Med. 1970;282:668–675. 4915800 10.1056/NEJM197003192821209

[20] Browning JD , Baxter J, Satapati S, et al The effect of short-term fasting on liver and skeletal muscle lipid, glucose, and energy metabolism in healthy women and men. J Lipid Res. 2012;53:577–586. 22140269 10.1194/jlr.P020867 PMC3276482

[21] Harvie MN , Pegington M, Mattson MP, et al The effects of intermittent or continuous energy restriction on weight loss and metabolic disease risk markers: a randomized trial in young overweight women. Int J Obes. 2011;35:714–727. 10.1038/ijo.2010.171 PMC3017674 20921964

[22] Gabel K , Kroeger CM, Trepanowski JF, et al Differential effects of alternate-day fasting versus daily calorie restriction on insulin resistance. Obesity (Silver Spring, Md) 2019;27:1443–1450. 31328895 10.1002/oby.22564 PMC7138754

[23] Weyer C , Bogardus C, Mott DM, et al The natural history of insulin secretory dysfunction and insulin resistance in the pathogenesis of type 2 diabetes mellitus. J Clin Invest. 1999;104:787–794. 10491414 10.1172/JCI7231 PMC408438

[24] Kitabchi AE , Temprosa M, Knowler WC, et al Role of insulin secretion and sensitivity in the evolution of type 2 diabetes in the diabetes prevention program: effects of lifestyle intervention and metformin. Diabetes 2005;54:2404–2414. 16046308 10.2337/diabetes.54.8.2404 PMC1360738

[25] Tabák AG , Jokela M, Akbaraly TN, et al Trajectories of glycaemia, insulin sensitivity, and insulin secretion before diagnosis of type 2 diabetes: an analysis from the Whitehall II study. Lancet. 2009;373:2215–2221. 19515410 10.1016/S0140-6736(09)60619-X PMC2726723

[26] Muoio DM , Newgard CB. Molecular and metabolic mechanisms of insulin resistance and β-cell failure in type 2 diabetes. Nat Rev Mol Cell Biol. 2008;9:193–205. 18200017 10.1038/nrm2327

[27] Haffner SM , D'Agostino R Jr, Mykkänen L, et al Insulin sensitivity in subjects with type 2 diabetes. Relationship to cardiovascular risk factors: the Insulin Resistance Atherosclerosis Study. Diabetes Care 1999;22:562–568. 10189532 10.2337/diacare.22.4.562

[28] Gast KB , Tjeerdema N, Stijnen T, et al Insulin resistance and risk of incident cardiovascular events in adults without diabetes: meta-analysis. PLoS One 2012;7: E 52036. 10.1371/journal.pone.0052036 PMC3532497 23300589

[29] Albosta M , Bakke J. Intermittent fasting: is there a role in the treatment of diabetes? A review of the literature and guide for primary care physicians. Clin Diabetes Endocrinol 2021;7:3. 33531076 10.1186/s40842-020-00116-1 PMC7856758

[30] Ahmed N , Farooq J, Siddiqi HS, et al Impact of intermittent fasting on lipid profile–a quasi-randomized clinical trial. Front Nutr. 2021;7:596787. 33598473 10.3389/fnut.2020.596787 PMC7882512

[31] Meng H , Zhu L, Kord-Varkaneh H, et al Effects of intermittent fasting and energy-restricted diets on lipid profile: a systematic review and meta-analysis. Nutrition 2020;77:110801. 32428841 10.1016/j.nut.2020.110801

[32] Wilhelmi de Toledo F , Grundler F, Bergouignan A, et al Safety, health improvement and well-being during a 4 to 21-day fasting period in an observational study including 1422 subjects. PLoS One 2019;14:e0209353. 30601864 10.1371/journal.pone.0209353 PMC6314618

[33] Malinowski B , Zalewska K, Węsierska A, et al Intermittent fasting in cardiovascular disorders-an overview. Nutrients. 2019;11:673. 30897855 10.3390/nu11030673 PMC6471315

[34] Eshghinia S , Mohammadzadeh F. The effects of modified alternate-day fasting diet on weight loss and CAD risk factors in overweight and obese women. J Diabetes Metab Disord 2013;12:4. 23497604 10.1186/2251-6581-12-4 PMC3598220

[35] Merimee TJ , Fineberg ES. Starvation-induced alterations of circulating thyroid hormone concentrations in man. Metabolism. 1976;25:79–83. 1246209 10.1016/0026-0495(76)90162-1

[36] Mattson MP , Arumugam TV. Hallmarks of brain aging: adaptive and pathological modification by metabolic states. Cell Metab 2018;27:1176–1199. 29874566 10.1016/j.cmet.2018.05.011 PMC6039826

[37] Speakman JR , Mitchell SE. Caloric restriction. Mol Aspects Med 2011;32:159–221. 21840335 10.1016/j.mam.2011.07.001

[38] Montine TJ , Phelps CH, Beach TG, et al National Institute on Aging—Alzheimer's Association guidelines for the neuropathologic assessment of Alzheimer's disease: a practical approach. Acta Neuropathol. 2012;123:1–11. 22101365 10.1007/s00401-011-0910-3 PMC3268003

[39] Ryan NS , Rossor MN, Fox NC. Alzheimer's disease in the 100 years since Alzheimer's death. Brain. 2015;138:3816–3821. 26541346 10.1093/brain/awv316

[40] Bancher C , Brunner C, Lassmann H, et al Accumulation of abnormally phosphorylated tau precedes the formation of neurofibrillary tangles in Alzheimer's disease. Brain Res. 1989;477:90–99. 2495152 10.1016/0006-8993(89)91396-6

[41] Bloom GS. Amyloid-β and tau: the trigger and bullet in Alzheimer disease pathogenesis. JAMA Neurol 2014;71:505–508. 24493463 10.1001/jamaneurol.2013.5847 PMC12908160

[42] Mawuenyega KG , Sigurdson W, Ovod V, et al Decreased clearance of CNS beta-amyloid in Alzheimer's disease. Science. 2010;330:1774. 21148344 10.1126/science.1197623 PMC3073454

[43] Wildsmith KR , Holley M, Savage JC, et al Evidence for impaired amyloid β clearance in Alzheimer's disease. Alzheimers Res Ther. 2013;5:33–33. 23849219 10.1186/alzrt187 PMC3978761

[44] Neu SC , Pa J, Kukull W, et al Apolipoprotein E genotype and sex risk factors for Alzheimer disease: a meta-analysis. JAMA Neurol. 2017;74:1178–1189. 28846757 10.1001/jamaneurol.2017.2188 PMC5759346

[45] Yamazaki Y , Zhao N, Caulfield TR, et al Apolipoprotein E and Alzheimer disease: pathobiology and targeting strategies. Nat Rev Neurol. 2019;15:501–518. 31367008 10.1038/s41582-019-0228-7 PMC7055192

[46] Castellano JM , Kim J, Stewart FR, et al Human apoE isoforms differentially regulate brain amyloid-β peptide clearance. Sci Transl Med. 2011;3:89ra57. 10.1126/scitranslmed.3002156 PMC3192364 21715678

[47] Breteler MMB. Vascular risk factors for Alzheimer’s disease: an epidemiologic perspective. Neurobiol Aging. 2000;21:153–160. 10867200 10.1016/s0197-4580(99)00110-4

[48] Lee H , Kim K, Lee YC, et al Associations between vascular risk factors and subsequent Alzheimer’s disease in older adults. Alzheimers Res Ther. 2020;12:117. 32979926 10.1186/s13195-020-00690-7 PMC7520023

[49] Zlokovic BV. Neurovascular pathways to neurodegeneration in Alzheimer's disease and other disorders. Nat Rev Neurosci. 2011;12:723–738. 22048062 10.1038/nrn3114 PMC4036520

[50] Iadecola C. The neurovascular unit coming of age: a journey through neurovascular coupling in health and disease. Neuron. 2017;96:17–42. 28957666 10.1016/j.neuron.2017.07.030 PMC5657612

[51] Zlokovic BV. Clearing amyloid through the blood-brain barrier. J Neurochem. 2004;89:807–811. 15140180 10.1111/j.1471-4159.2004.02385.x

[52] Gosselet F , Candela P, Cecchelli R, et al Role of the blood-brain barrier in Alzheimer's disease. Med Sci (Paris) 2011;27:987–992. 22130026 10.1051/medsci/20112711015

[53] Cockerill I , Oliver JA, Xu H, et al Blood-brain barrier integrity and clearance of amyloid-β from the BBB. Adv Exp Med Biol. 2018;1097:261–278. 30315550 10.1007/978-3-319-96445-4_14

[54] Starr JM , Farrall AJ, Armitage P, et al Blood–brain barrier permeability in Alzheimer's disease: a case–control MRI study. Psychiatry Res Neuroimaging 2009;171:232–241. 10.1016/j.pscychresns.2008.04.003 19211227

[55] Wardlaw JM , Makin SJ, Valdés Hernández MC, et al Blood-brain barrier failure as a core mechanism in cerebral small vessel disease and dementia: evidence from a cohort study. Alzheimer's Dement 2017;13:634–643.

[56] de la Torre JC. The vascular hypothesis of Alzheimer’s disease: bench to bedside and beyond. Neurodegenerat Dis. 2010;7:116–121. 10.1159/000285520 20173340

[57] Roher AE , Debbins JP, Malek-Ahmadi M, et al Cerebral blood flow in Alzheimer's disease. Vasc Health Risk Manag. 2012;8:599–611. 23109807 10.2147/VHRM.S34874 PMC3481957

[58] Montagne A , Barnes SR, Sweeney MD, et al Blood-brain barrier breakdown in the aging human hippocampus. Neuron. 2015;85:296–302. 25611508 10.1016/j.neuron.2014.12.032 PMC4350773

[59] Yang AC , Vest RT, Kern F, et al A human brain vascular atlas reveals diverse mediators of Alzheimer’s risk. Nature. 2022;603:885–892. 35165441 10.1038/s41586-021-04369-3 PMC9635042

[60] Rogers J , Luber-Narod J, Styren SD, et al Expression of immune system-associated antigens by cells of the human central nervous system: relationship to the pathology of Alzheimer's disease. Neurobiol Aging. 1988;9:339–349. 3263583 10.1016/s0197-4580(88)80079-4

[61] Bradburn S , Murgatroyd C, Ray N. Neuroinflammation in mild cognitive impairment and Alzheimer's disease: a meta-analysis. Ageing Res Rev. 2019;50:1–8. 30610927 10.1016/j.arr.2019.01.002

[62] Kinney JW , Bemiller SM, Murtishaw AS, et al Inflammation as a central mechanism in Alzheimer's disease. Alzheimers Dement (N Y) 2018;4:575–590. 30406177 10.1016/j.trci.2018.06.014 PMC6214864

[63] Mrak RE , Sheng JG, Griffin WS. Glial cytokines in Alzheimer's disease: review and pathogenic implications. Hum Pathol. 1995;26:816–823. 7635444 10.1016/0046-8177(95)90001-2 PMC3903413

[64] Griffin WS , Stanley LC, Ling C, et al Brain interleukin 1 and S-100 immunoreactivity are elevated in Down syndrome and Alzheimer disease. Proc Natl Acad Sci USA. 1989;86:7611–7615. 2529544 10.1073/pnas.86.19.7611 PMC298116

[65] Liu B , Le KX, Park MA, et al In Vivo detection of age- and disease-related increases in neuroinflammation by 18F-GE180 TSPO MicroPET imaging in wild-type and Alzheimer's transgenic mice. J Neurosci 2015;35:15716–15730. 26609163 10.1523/JNEUROSCI.0996-15.2015 PMC6705465

[66] Cribbs DH , Berchtold NC, Perreau V, et al Extensive innate immune gene activation accompanies brain aging, increasing vulnerability to cognitive decline and neurodegeneration: a microarray study. J Neuroinflamm. 2012;9:179–179. 10.1186/1742-2094-9-179 PMC3419089 22824372

[67] Gomez-Nicola D , Boche D. Post-mortem analysis of neuroinflammatory changes in human Alzheimer's disease. Alzheimers Res Ther. 2015;7:42. 25904988 10.1186/s13195-015-0126-1 PMC4405851

[68] Ismail R , Parbo P, Madsen LS, et al The relationships between neuroinflammation, beta-amyloid and tau deposition in Alzheimer’s disease: a longitudinal PET study. J Neuroinflammation 2020;17:151. 32375809 10.1186/s12974-020-01820-6 PMC7203856

[69] Kolb H , Kempf K, Röhling M, et al Ketone bodies: from enemy to friend and guardian angel. BMC Med. 2021;19:313. 34879839 10.1186/s12916-021-02185-0 PMC8656040

[70] Kashiwaya Y , Takeshima T, Mori N, et al d-beta-hydroxybutyrate protects neurons in models of Alzheimer's and Parkinson's disease. Proc Natl Acad Sci USA. 2000;97:5440–5444. 10805800 10.1073/pnas.97.10.5440 PMC25847

[71] Versele R , Corsi M, Fuso A, et al Ketone bodies promote amyloid-β(1-40) clearance in a human in vitro blood-brain barrier model. Int J Mol Sci 2020;21:934. 32023814 10.3390/ijms21030934 PMC7037612

[72] Halagappa VK , Guo Z, Pearson M, et al Intermittent fasting and caloric restriction ameliorate age-related behavioral deficits in the triple-transgenic mouse model of Alzheimer's disease. Neurobiol Dis 2007;26:212–220. 17306982 10.1016/j.nbd.2006.12.019

[73] Zhang J , Zhan Z, Li X, et al Intermittent fasting protects against Alzheimer's disease possible through restoring aquaporin-4 polarity. Front Mol Neurosci. 2017;10:395. 29238290 10.3389/fnmol.2017.00395 PMC5712566

[74] Kashiwaya Y , Bergman C, Lee J-H, et al A ketone ester diet exhibits anxiolytic and cognition-sparing properties, and lessens amyloid and tau pathologies in a mouse model of Alzheimer's disease. Neurobiol Aging. 2013;34:1530–1539. 23276384 10.1016/j.neurobiolaging.2012.11.023 PMC3619192

[75] Arumugam TV , Phillips TM, Cheng A, et al Age and energy intake interact to modify cell stress pathways and stroke outcome. Ann Neurol. 2010;67:41–52. 20186857 10.1002/ana.21798 PMC2844782

[76] Lazic D , Tesic V, Jovanovic M, et al Every-other-day feeding exacerbates inflammation and neuronal deficits in 5XFAD mouse model of Alzheimer's disease. Neurobiol Dis. 2020;136:104745. 31931140 10.1016/j.nbd.2020.104745

[77] Shin BK , Kang S, Kim DS, et al Intermittent fasting protects against the deterioration of cognitive function, energy metabolism and dyslipidemia in Alzheimer's disease-induced estrogen deficient rats. Exp Biol Med (Maywood). 2018;243:334–343. 29307281 10.1177/1535370217751610 PMC6022926

[78] Hu X , De Silva TM, Chen J, et al Cerebral vascular disease and neurovascular injury in ischemic stroke. Circ Res. 2017;120:449–471. 28154097 10.1161/CIRCRESAHA.116.308427 PMC5313039

[79] Tuttolomondo A , Daidone M, Pinto A. Endothelial dysfunction and inflammation in ischemic stroke pathogenesis. Curr Pharm Des. 2020;26:4209–4219. 32303167 10.2174/1381612826666200417154126

[80] Esmaeilzadeh N , van de Borne P. Does intermittent fasting improve microvascular endothelial function in healthy middle-aged subjects? Biol Med (Aligarh). 2016;8:6.

[81] Yousefi B , Faghfoori Z, Samadi N, et al The effects of Ramadan fasting on endothelial function in patients with cardiovascular diseases. Eur J Clin Nutr. 2014;68:835–839. 24755927 10.1038/ejcn.2014.61

[82] Sun J , Zhang T, Zhang L, et al Fasting therapy contributes to the improvement of endothelial function and decline in vascular injury-related markers in overweight and obese individuals via activating autophagy of endothelial progenitor cells. Evid-Based Complement Alternat Med. 2020;2020:3576030. 32802124 10.1155/2020/3576030 PMC7403908

[83] Xin B , Liu CL, Yang H, et al Prolonged fasting improves endothelial progenitor cell-mediated ischemic angiogenesis in mice. Cell Physiol Biochem. 2016;40:693–706. 27898404 10.1159/000452581

[84] Jeong JH , Yu KS, Bak DH, et al Intermittent fasting is neuroprotective in focal cerebral ischemia by minimizing autophagic flux disturbance and inhibiting apoptosis. Exp Ther Med. 2016;12:3021–3028. 27882110 10.3892/etm.2016.3852 PMC5103738

[85] Fann DY , Ng GY, Poh L, et al Positive effects of intermittent fasting in ischemic stroke. Exp Gerontol. 2017;89:93–102. 28115234 10.1016/j.exger.2017.01.014

[86] Santos CY , Snyder PJ, Wu W-C, et al Pathophysiologic relationship between Alzheimer's disease, cerebrovascular disease, and cardiovascular risk: a review and synthesis. Alzheimer's Dement. 2017;7:69–87. 10.1016/j.dadm.2017.01.005 PMC5328683 28275702

[87] Mattson MP , Wan R. Beneficial effects of intermittent fasting and caloric restriction on the cardiovascular and cerebrovascular systems. J Nutr Biochem. 2005;16:129–137. 15741046 10.1016/j.jnutbio.2004.12.007

[88] Goldenberg I , Goldkorn R, Shlomo N, et al Heart rate variability for risk assessment of myocardial ischemia in patients without known coronary artery disease: the HRV-DETECT (Heart Rate Variability for the Detection of Myocardial Ischemia) Study. J Am Heart Assoc. 2019;8:e014540. 31838969 10.1161/JAHA.119.014540 PMC6951049

[89] Kjeldsen-Kragh J , Haugen M, Borchgrevink CF, et al Controlled trial of fasting and one-year vegetarian diet in rheumatoid arthritis. Lancet. 1991;338:899–902. 1681264 10.1016/0140-6736(91)91770-u

[90] Müller FWdTKLR H. Fasting followed by vegetarian diet in patients with rheumatoid arthritis: a systematic review. Scand J Rheumatol. 2001;30:1–10. 11252685 10.1080/030097401750065256

[91] Pedditizi E , Peters R, Beckett N. The risk of overweight/obesity in mid-life and late life for the development of dementia: a systematic review and meta-analysis of longitudinal studies. Age Ageing. 2016;45:14–21. 26764391 10.1093/ageing/afv151

[92] Kivimäki M , Luukkonen R, Batty GD, et al Body mass index and risk of dementia: analysis of individual-level data from 1.3 million individuals. Alzheimers Dement. 2018;14:601–609. 29169013 10.1016/j.jalz.2017.09.016 PMC5948099

[93] Strazzullo P , D'Elia L, Cairella G, et al Excess body weight and incidence of stroke. Stroke 2010;41:e418–e426. 20299666 10.1161/STROKEAHA.109.576967

[94] Van Gaal LF , Mertens IL, De Block CE. Mechanisms linking obesity with cardiovascular disease. Nature. 2006;444:875–880. 17167476 10.1038/nature05487

[95] Patikorn C , Roubal K, Veettil SK, et al Intermittent fasting and obesity-related health outcomes: an umbrella review of meta-analyses of randomized clinical trials. JAMA Netw Open. 2021;4:e2139558. 34919135 10.1001/jamanetworkopen.2021.39558 PMC8683964

[96] Faris MA , Kacimi S, Al-Kurd RA, et al Intermittent fasting during Ramadan attenuates proinflammatory cytokines and immune cells in healthy subjects. Nutr Res. 2012;32:947–955. 23244540 10.1016/j.nutres.2012.06.021

[97] Jordan S , Tung N, Casanova-Acebes M, et al Dietary intake regulates the circulating inflammatory monocyte pool. Cell. 2019;178:1102–1114.e1117. 31442403 10.1016/j.cell.2019.07.050 PMC7357241

[98] Horne BD , Anderson JL, May HT, et al Intermittent fasting and changes in galectin-3: a secondary analysis of a randomized controlled trial of disease-free subjects. Nutr Metab Cardiovasc Dis. 2022;32:1538–1548. 35361560 10.1016/j.numecd.2022.03.001

[99] Singh R , Lakhanpal D, Kumar S, et al Late-onset intermittent fasting dietary restriction as a potential intervention to retard age-associated brain function impairments in male rats. Age (Dordr). 2012;34:917–933. 21861096 10.1007/s11357-011-9289-2 PMC3682068

[100] Fontán-Lozano A , Sáez-Cassanelli JL, Inda MC, et al Caloric restriction increases learning consolidation and facilitates synaptic plasticity through mechanisms dependent on NR2B subunits of the NMDA receptor. J Neurosci. 2007;27:10185–10195. 17881524 10.1523/JNEUROSCI.2757-07.2007 PMC6672666

[101] Lee J , Seroogy KB, Mattson MP. Dietary restriction enhances neurotrophin expression and neurogenesis in the hippocampus of adult mice. J Neurochem. 2002;80:539–547. 11905999 10.1046/j.0022-3042.2001.00747.x

[102] Li L , Wang Z, Zuo Z. Chronic intermittent fasting improves cognitive functions and brain structures in mice. PLoS One. 2013;8:e66069. 23755298 10.1371/journal.pone.0066069 PMC3670843

[103] Qasrawi SO , Pandi-Perumal SR, BaHammam AS. The effect of intermittent fasting during Ramadan on sleep, sleepiness, cognitive function, and circadian rhythm. Sleep Breath. 2017;21:577–586. 28190167 10.1007/s11325-017-1473-x

[104] Tian H-H , Aziz A-R, Png W, et al Effects of fasting during Ramadan month on cognitive function in Muslim athletes. Asian J Sports Med. 2011;2:145–153. 22375233 10.5812/asjsm.34753 PMC3289210

[105] BaHammam AS , Nashwan S, Hammad O, et al Objective assessment of drowsiness and reaction time during intermittent Ramadan fasting in young men: a case-crossover study. Behav Brain Funct. 2013;9:32. 23937904 10.1186/1744-9081-9-32 PMC3751553

[106] Ghayour Najafabadi M , Rahbar Nikoukar L, Memari A, et al Does Ramadan fasting adversely affect cognitive function in young females? Scientifica (Cairo). 2015;2015:432428. 26697263 10.1155/2015/432428 PMC4677254

[107] Benau EM , Orloff NC, Janke EA, et al A systematic review of the effects of experimental fasting on cognition. Appetite. 2014;77:52–61. 24583414 10.1016/j.appet.2014.02.014

[108] Green MW , Elliman NA, Rogers PJ. Lack of effect of short-term fasting on cognitive function. J Psychiatr Res. 1995;29:245–253. 7473300 10.1016/0022-3956(95)00009-t

[109] Witte AV , Fobker M, Gellner R, et al Caloric restriction improves memory in elderly humans. Proc Natl Acad Sci USA. 2009;106:1255–1260. 19171901 10.1073/pnas.0808587106 PMC2633586

[110] Leclerc E , Trevizol AP, Grigolon RB, et al The effect of caloric restriction on working memory in healthy non-obese adults. CNS Spectr. 2020;25:2–8. 30968820 10.1017/S1092852918001566

[111] Ooi TC , Meramat A, Rajab NF, et al Intermittent fasting enhanced the cognitive function in older adults with mild cognitive impairment by inducing biochemical and metabolic changes: a 3-year progressive study. Nutrients. 2020;12:2644. 32872655 10.3390/nu12092644 PMC7551340

[112] Reger MA , Henderson ST, Hale C, et al Effects of beta-hydroxybutyrate on cognition in memory-impaired adults. Neurobiol Aging. 2004;25:311–314. 15123336 10.1016/S0197-4580(03)00087-3

[113] Jensen NJ , Wodschow HZ, Nilsson M, et al Effects of ketone bodies on brain metabolism and function in neurodegenerative diseases. Int J Mol Sci. 2020;21:8767. 33233502 10.3390/ijms21228767 PMC7699472

[114] Cioffi I , Evangelista A, Ponzo V, et al Intermittent versus continuous energy restriction on weight loss and cardiometabolic outcomes: a systematic review and meta-analysis of randomized controlled trials. J Transl Med. 2018;16:371–371. 30583725 10.1186/s12967-018-1748-4 PMC6304782

[115] Grajower MM , Horne BD. Clinical management of intermittent fasting in patients with diabetes mellitus. Nutrients. 2019;11:873. 31003482 10.3390/nu11040873 PMC6521152

[116] Mattson MP , Cutler RG, Camandola S. Energy intake and amyotrophic lateral sclerosis. Neuromol Med. 2007;9:17–20. 10.1385/nmm:9:1:17 17114821

[117] Pedersen WA , Mattson MP. No benefit of dietary restriction on disease onset or progression in amyotrophic lateral sclerosis Cu/Zn-superoxide dismutase mutant mice. Brain Res. 1999;833:117–120. 10375685 10.1016/s0006-8993(99)01471-7

[118] Lowe DA , Wu N, Rohdin-Bibby L, et al Effects of time-restricted eating on weight loss and other metabolic parameters in women and men with overweight and obesity: the TREAT randomized clinical trial. JAMA Intern Med. 2020;180:1491–1499. 32986097 10.1001/jamainternmed.2020.4153 PMC7522780

[119] Chow LS , Manoogian ENC, Alvear A, et al Time-restricted eating effects on body composition and metabolic measures in humans who are overweight: a feasibility study. Obesity (Silver Spring). 2020;28:860–869. 10.1002/oby.22756 PMC7180107 32270927

[120] Tinsley GM , Forsse JS, Butler NK, et al Time-restricted feeding in young men performing resistance training: a randomized controlled trial. Eur J Sport Sci. 2017;17:200–207. 27550719 10.1080/17461391.2016.1223173

[121] Verreijen AM , Verlaan S, Engberink MF, et al A high whey protein-, leucine-, and vitamin D-enriched supplement preserves muscle mass during intentional weight loss in obese older adults: a double-blind randomized controlled trial. Am J Clin Nutr. 2015;101:279–286. 25646324 10.3945/ajcn.114.090290

[122] Kim JE , O'Connor LE, Sands LP, et al Effects of dietary protein intake on body composition changes after weight loss in older adults: a systematic review and meta-analysis. Nutr Rev. 2016;74:210–224. 26883880 10.1093/nutrit/nuv065 PMC4892287

[123] Miyamoto K , Higashino S, Mochizuki K, et al Evaluation of weight loss in the community-dwelling elderly with dementia as assessed by eating behavior and mental status. Asia Pac J Clin Nutr. 2011;20:9–13. 21393104

[124] Allaf M , Elghazaly H, Mohamed OG, et al Intermittent fasting for the prevention of cardiovascular disease. Cochrane Database Syst Rev 2021;(1):CD013496. 33512717 10.1002/14651858.CD013496.pub2 PMC8092432

[125] Liu D , Huang Y, Huang C, et al Calorie restriction with or without time-restricted eating in weight loss. New Engl J Med 2022;386:1495–1504. 35443107 10.1056/NEJMoa2114833

[126] Nasaruddin ML , Syed Abd Halim SA, Kamaruzzaman MA. Studying the relationship of intermittent fasting and β-amyloid in animal model of Alzheimer’s disease: a scoping review. Nutrients. 2020;12:3215. 33096730 10.3390/nu12103215 PMC7590153

[127] Pannen ST , Maldonado SG, Nonnenmacher T, et al Adherence and dietary composition during intermittent vs. continuous calorie restriction: follow-up data from a randomized controlled trial in adults with overweight or obesity. Nutrients. 2021;13:1195. 33916366 10.3390/nu13041195 PMC8067073

[128] Trepanowski JF , Kroeger CM, Barnosky A, et al Effect of alternate-day fasting on weight loss, weight maintenance, and cardioprotection among metabolically healthy obese adults: a randomized clinical trial. JAMA Intern Med. 2017;177:930–938. 28459931 10.1001/jamainternmed.2017.0936 PMC5680777

[129] Lemstra M , Bird Y, Nwankwo C, et al Weight loss intervention adherence and factors promoting adherence: a meta-analysis. Patient Prefer Adherence. 2016;10:1547–1559. 27574404 10.2147/PPA.S103649 PMC4990387

[130] Anton SD , Lee SA, Donahoo WT, et al The effects of time restricted feeding on overweight, older adults: a pilot study. Nutrients. 2019;11:1500. 31262054 10.3390/nu11071500 PMC6682944

[131] Domaszewski P , Konieczny M, Pakosz P, et al Effect of a six-week intermittent fasting intervention program on the composition of the human body in women over 60 years of age. Int J Environ Res Public Health. 2020;17:4138. 32531956 10.3390/ijerph17114138 PMC7312819

[132] Conley M, L , Fevre L, Haywood C, et al Is two days of intermittent energy restriction per week a feasible weight loss approach in obese males? A randomised pilot study. Nutr Diet. 2018;75:65–72. 28791787 10.1111/1747-0080.12372

[133] Shi J , Sabbagh MN, Vellas B. Alzheimer's disease beyond amyloid: strategies for future therapeutic interventions. BMJ. 2020;371: M 3684. 10.1136/bmj.m3684 PMC7541037 33036984

[134] Miller MB , Huang AY, Kim J, et al Somatic genomic changes in single Alzheimer’s disease neurons. Nature. 2022;604:714–722. 35444284 10.1038/s41586-022-04640-1 PMC9357465

[135] Butterfield DA , Halliwell B. Oxidative stress, dysfunctional glucose metabolism and Alzheimer disease. Nat Rev Neurosci. 2019;20:148–160. 30737462 10.1038/s41583-019-0132-6 PMC9382875

[136] Horne BD , Muhlestein JB, May HT, et al Relation of routine, periodic fasting to risk of diabetes mellitus, and coronary artery disease in patients undergoing coronary angiography. Am J Cardiol. 2012;109:1558–1562. 22425331 10.1016/j.amjcard.2012.01.379

[137] Ganson KT , Rodgers RF, Murray SB, et al Prevalence and demographic, substance use, and mental health correlates of fasting among U.S. college students. J Eating Disord. 2021;9:88. 10.1186/s40337-021-00443-3 PMC8293526 34289904

[138] Ballard J. Americans say this popular diet is effective and inexpensive. YouGovAmerica website. February 24, 2020. Available at: https://today.yougov.com/topics/consumer/articles-reports/2020/02/24/most-effective-diet-intermittent-fasting-poll. Accessed March 1, 2023.

[139] Midena K. From 5:2 to 16:8, do intermittent fasting diets work, and what are the challenges? ABC Science website. Updated August 3, 2021. Available at: https://amp.abc.net.au/article/100315506. Accessed March 1, 2023.

